# Macrophage-mediated inflammation and microcalcification are associated with abdominal aortic aneurysm progression and clinical events

**DOI:** 10.1093/bjs/znag100

**Published:** 2026-08-12

**Authors:** Rachael O Forsythe, Olivia M B McBride, Jennifer M J Robson, Alex T Vesey, Allison C Winarski, Callum Weston, Samuel Debono, Scott I Semple, Calum D Gray, Thomas J MacGillivray, Julie Brittenden, Giles Roditi, Wesley Stuart, Edwin J R van Beek, Andrew L Tambyraja, Michelle C Williams, Marc R Dweck, David E Newby

**Affiliations:** Edinburgh Vascular Unit, Royal Infirmary of Edinburgh, Edinburgh, UK; Centre for Cardiovascular Science, University of Edinburgh, Edinburgh, UK; Ninewells Vascular Unit, Ninewells Hospital, Dundee, UK; Leeds Vascular Institute, Leeds General Infirmary, Leeds, UK; Department of Vascular Surgery, University Hospital Hairmyres, Glasgow, UK; Edinburgh Vascular Unit, Royal Infirmary of Edinburgh, Edinburgh, UK; Centre for Cardiovascular Science, University of Edinburgh, Edinburgh, UK; Edinburgh Vascular Unit, Royal Infirmary of Edinburgh, Edinburgh, UK; Department of Vascular Surgery, Queen Elizabeth University Hospital, Glasgow, UK; Edinburgh Imaging Facility, Queen’s Medical Research Institute, University of Edinburgh, Edinburgh, UK; Edinburgh Imaging Facility, Queen’s Medical Research Institute, University of Edinburgh, Edinburgh, UK; Edinburgh Imaging Facility, Queen’s Medical Research Institute, University of Edinburgh, Edinburgh, UK; Department of Vascular Surgery, Queen Elizabeth University Hospital, Glasgow, UK; School of Cardiovascular & Metabolic Health, Glasgow Cardiovascular Research Centre, Glasgow, UK; School of Cardiovascular & Metabolic Health, Glasgow Cardiovascular Research Centre, Glasgow, UK; Edinburgh Imaging Facility, Queen’s Medical Research Institute, University of Edinburgh, Edinburgh, UK; Edinburgh Vascular Unit, Royal Infirmary of Edinburgh, Edinburgh, UK; Centre for Cardiovascular Science, University of Edinburgh, Edinburgh, UK; Centre for Cardiovascular Science, University of Edinburgh, Edinburgh, UK; Centre for Cardiovascular Science, University of Edinburgh, Edinburgh, UK

## Abstract

**Background:**

Molecular and cellular imaging biomarkers of abdominal aortic aneurysms (AAAs) show promise as potential predictors of clinical events. This study assessed long-term clinical outcomes using ultrasmall superparamagnetic particles of iron oxide (USPIO)-enhanced MRI (ISRCTN76413758) and fluorine-18 sodium fluoride ([^18^F]NaF) PET/CT (NCT02229006).

**Methods:**

In two prospective multicentre co-enrolled observational cohort studies of patients with asymptomatic AAAs, evaluation of aortic wall inflammation using USPIO-enhanced MRI in 338 patients was followed by [^18^F]NaF PET/CT in a subgroup of 72 patients, to evaluate microcalcification. Aneurysms were classified according to aortic wall USPIO enhancement and into tertiles of [^18^F]NaF uptake. Aneurysm expansion, composite of rupture or repair, and overall survival were compared.

**Results:**

In 338 predominantly elderly (mean(s.d.) age of 73.5(7.2) years) men (85.2%) with a mean(s.d.) aneurysm diameter of 49.5(7.3) mm, aneurysm expansion was increased in patients with USPIO enhancement (median of 3.3 (interquartile range (i.q.r.) 2.0–4.6) *versus* 2.5 (i.q.r. 1.4–3.6) mm/year; *P* = 0.002) or high [^18^F]NaF uptake (median of 4.0 (i.q.r. 2.6–5.4) *versus* 1.9 (i.q.r. 0.9–3.0) mm/year; *P* < 0.001), even when adjusted for hypertension, smoking, and baseline AAA diameter (*P* = 0.038 and *P* = 0.003). After a median follow-up of 102 (i.q.r. 58–119) months, 62.7% of patients met the composite endpoint of rupture or repair, with high [^18^F]NaF uptake associated with a more than two-fold higher risk (72% *versus* 36%; log rank *P* = 0.002). There was no association between USPIO enhancement and future rupture or repair.

**Conclusion:**

Aortic wall USPIO enhancement and [^18^F]NaF uptake are independently associated with AAA expansion. These non-invasive imaging biomarkers provide an opportunity to identify those at the greatest risk of AAA progression and may help inform further research to determine optimal surveillance intervals.

## Teaser

In patients with asymptomatic abdominal aortic aneurysms (AAAs) under surveillance, future growth rate or rupture risk is difficult to determine on an individual patient basis. The use of novel imaging techniques to identify aneurysms with increased inflammation and microcalcification, two key processes implicit in aneurysm disease, can identify those at the greatest risk of aneurysm expansion. Non-invasive molecular and cellular imaging techniques may be useful to determine patients with an increased risk of AAA expansion and may therefore help decide whether or when to intervene. These imaging techniques may have other potential translational roles including quantifying disease response to novel therapies.

## Introduction

Molecular and cellular imaging biomarkers have been explored in patients with abdominal aortic aneurysms (AAAs) in the hope of providing non-invasive, patient-specific prediction of future disease progression^1^. Ultrasmall superparamagnetic particles of iron oxide (USPIO) can be used to identify tissue-resident macrophage activity^2^ and this agent has also been explored as a cellular imaging biomarker of inflammation in several cardiovascular diseases, including the carotid arteries following stroke and the myocardium following myocardial infarction^3–5^. A prospective observational cohort study of 342 patients with asymptomatic AAAs under surveillance followed up for 2.7 years has previously shown that high aortic wall accumulation of USPIO (a marker of macrophage-mediated inflammation)^6^ evaluated using MRI is associated with future aneurysm expansion in the medium-term^7^.

Molecular processes within the arterial wall can be evaluated using target-specific radiotracers. Whilst the most widely used radiotracer fluorine-18 fluorodeoxyglucose ([^18^F]FDG) may identify arterial inflammation in many arterial beds, results using this tracer have been conflicting in AAA disease^8–10^. Fluorine-18 sodium fluoride ([^18^F]NaF) is an alternative PET radiotracer that provides a marker of developing vascular microcalcification that has been associated with clinical events including myocardial infarction^11^, progression of aortic valve disease^12^, re-stenosis following lower limb angioplasty^13^, and stroke^14^. In AAAs, microcalcification may be a marker of inflammation, vascular smooth muscle cell (VSMC) apoptosis, and aortic wall degradation. In a substudy of 72 patients co-enrolled in a USPIO MRI study, high [^18^F]NaF uptake on PET/CT (representing increased microcalcification) was associated with short-term aneurysm expansion and risk of AAA rupture or repair in the short-term, independent of traditional risk factors such as age, sex, systolic blood pressure, smoking, and aneurysm diameter^15^.

The aim of this study was to report the long-term outcomes of patients with AAAs who had previously undergone molecular ([^18^F]NaF PET/CT to identify microcalcification) and cellular (USPIO MRI to quantify macrophage-mediated inflammation) imaging as part of two co-enrolled clinical studies, evaluating two distinct pathobiological processes implicated in aneurysm disease.

## Methods

### Study population

Patients aged >50 years who were participants in the MA^3^RS (MRI using Ultrasmall Superparamagnetic Particles of Iron Oxide to Predict Clinical Outcomes in Patients Under Surveillance for Abdominal Aortic Aneurysms; ISRCTN76413758)^16^ and SoFIA^3^ (Sodium Fluoride Imaging of Abdominal Aortic Aneurysms; NCT02229006)^15^ studies were eligible for inclusion. Participants were excluded if they had withdrawn from follow-up for these studies or, as previously described^7,15^, had any contraindications to iodinated contrast or imaging probe administration, were pregnant, or if they had a known connective tissue disorder or vasculitis.

### Study design

The MA^3^RS study was a multicentre observational open-label cohort study of patients under routine surveillance for AAAs with an anteroposterior diameter ≥40 mm according to ultrasonography, who underwent USPIO MRI at baseline to determine the presence of USPIO accumulation in the aortic wall, representative of macrophage-mediated inflammation. The SoFIA^3^ study was a substudy of the MA^3^RS study, whereby patients underwent PET/CT to determine uptake of aortic wall [^18^F]NaF, a marker of microcalcification. Both studies were conducted with written consent of all patients and approved by the East of Scotland (MA^3^RS: 12/ES/0068) and South East Scotland (SoFIA^3^: 14/SS/0080) Research Ethics Committees. The studies are reported according to the STROBE statement^17^.

### Study assessments

In the MA^3^RS study, aortic wall inflammation was evaluated using USPIO-enhanced MRI. Patients underwent baseline 3T MRI (Magnetom Verio 3T, Siemens Healthineers, Erlangen, Germany) and CT angiography before receiving an intravenous infusion of a weight-adjusted dose of USPIO (4 mg/kg ferumoxytol; Rienso, Takeda UK, London, UK). A second MRI scan was performed 24–36 h after USPIO administration. Bespoke software was used to perform image analysis, generating colour maps to depict the change in T2* (the decay constant for the exponential decay of signal over time) after USPIO administration. Scans were classified by two independent trained observers (O.M.B.M. and R.O.F.) as USPIO positive if ten contiguous voxels of USPIO enhancement were identified in the aortic wall (representing tissue-resident aortic wall macrophage activity) or USPIO negative if not (*Fig. 1*). This method of image analysis has been published previously^7,16^.

**Figure 1:**
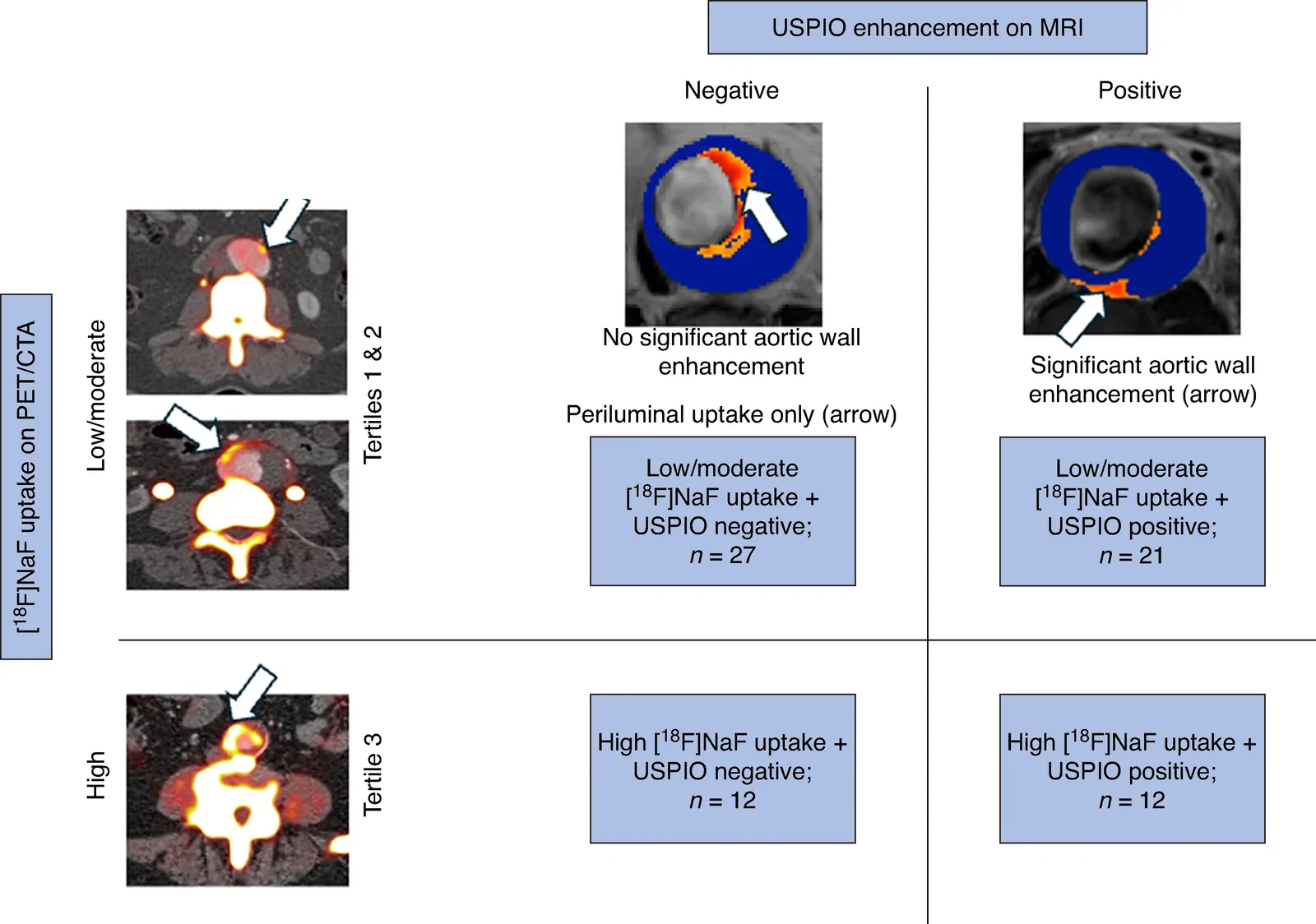
Classification of aneurysms according to aortic wall UPSIO enhancement on MRI and [^18^F]NaF uptake on PET/CT USPIO, ultrasmall superparamagnetic particles of iron oxide; [^18^F]NaF, fluorine-18 sodium fluoride.

In the SoFIA^3^ study, aortic wall microcalcification was assessed using [^18^F]NaF PET/CT. Participants underwent low-dose attenuation correction CT, ECG-gated CT calcium scoring, CT angiography, and PET acquisition 60 min after intravenous administration of a target dose of 125 MBq [^18^F]NaF on a hybrid 128-detector array PET/CT scanner (Biograph mCT, Siemens). Aneurysms were categorized into tertiles of [^18^F]NaF uptake, by first quantifying the maximum standardized uptake value (SUV; a validated measure of tissue radiotracer uptake) in the regions of interest (consecutive axial slices encompassing the aortic wall and excluding overspill from adjacent bone structures) and calculating the maximum target-to-background ratio (TBRmax) by correcting maximum SUV for background blood pool activity, using the averaged mean SUV of three consecutive regions of interest in the right atrium. The ‘most diseased segment’ approach was used, as described previously and reported elsewhere^18^, by calculating the mean TBRmax across three axial slices centred on the region of the aneurysm sac with the highest tracer activity (*Fig. 1*). TBRmax values were log-transformed (log_2_) to normalize the data.

Ultrasonographic evaluation of the maximum anteroposterior aneurysm diameter was undertaken in an accredited vascular laboratory according to local surveillance protocols: every 12 months for aneurysms with an anteroposterior diameter <4 cm; every 6 months for aneurysms with an anteroposterior diameter 4–5 cm; and every 3 months for aneurysms with an anteroposterior diameter >5 cm. The vascular laboratory practitioners were independent of the study team.

### Clinical endpoints

The primary endpoint was the composite of aneurysm rupture or repair. Secondary endpoints were aneurysm expansion and overall survival including all-cause death, cardiovascular death, and aneurysm-related death. Structured follow-up data were collected on AAA events, hospital admissions, and other relevant clinical data by two independent observers, verified using electronic health records and through a search of public registry data to obtain death certificates. Clinicians were blinded to the results of the research scans; all decisions on the timing of surgery were made independent of the research study findings.

### Statistics and data analysis

Summary demographic data are reported as *n* (%) for categorical variables and mean(s.d.) or median (interquartile range (i.q.r.)) for continuous variables, as appropriate. The entire MA^3^RS study cohort (342 patients) was stratified as USPIO positive or USPIO negative to assess associations between USPIO enhancement and clinical outcomes. For the 72 patients who also underwent [^18^F]NaF PET/CT as part of the SoFIA^3^ study, classification was according to tertiles of [^18^F]NaF uptake in the most-diseased aneurysm segment and these patients were further categorized according to the combination of USPIO enhancement on MRI and [^18^F]NaF uptake on PET/CT (*Fig. 1*), as an exploratory analysis due to the small numbers in each classification group.

To investigate any association between USPIO enhancement, [^18^F]NaF uptake, or the combination of the two imaging biomarkers and future aneurysm expansion, Student’s *t* test or a Mann–Whitney test was used, as appropriate. One-way ANOVA was used to compare continuous variables across multiple factors, with post-hoc analysis using the Bonferroni test, where required. Linear regression was used to assess the independent association of biomarker category with aneurysm expansion (mm/year), adjusting for baseline size, hypertension, and current smoking. Categorial predictors (smoking, hypertension, biomarker group) were entered as factors and baseline aneurysm size as a continuous covariate. Collinearity diagnostics were checked to ensure model validity. Regression coefficients, 95% confidence intervals, *t* statistics, and *P* values are reported.

Kaplan-Meier analysis was used to assess time to the composite endpoint of AAA repair or rupture for each biomarker and for biomarker combinations, with distributions compared using log-rank test. Multivariable regression analysis was used to adjust for hypertension, current smoking, and baseline AAA diameter. Statistical analysis was undertaken using JASP version 0.95.4, with significance taken at the two-sided 5% level (*P* < 0.05). AAA repair and rupture were treated as components of the composite outcome and not as competing events.

## Results

The study population consisted of 342 patients in the MA^3^RS cohort, of whom 72 also underwent [^18^F]NaF PET/CT as part of the SoFIA^3^ study, with a median of 14.1 (i.q.r. 4.4–23.4] months between scans. The median follow-up duration was 102 (i.q.r. 58–119) months for the overall cohort and 101 (i.q.r. 63–108) months for the SoFIA^3^ substudy cohort. Follow-up data were available for 338 patients, with 4 participants lost to follow-up. As previously reported^7^, patients were predominantly elderly (mean(s.d.) age of 73.5 (7.2) years) men (288 (85.2%)) with expected cardiovascular risk factors including smoking history (289 (85.5%) current smokers or ex-smokers), hypertension (243 (71.9%)), and hypercholesterolaemia (256 (75.7%)). The mean(s.d.) aneurysm diameter at baseline was 49.5(7.3) mm and 17.8% of patients had a family history of AAAs.

USPIO enhancement was associated with smoking status and aneurysm diameter at baseline. [^18^F]NaF uptake was not associated with baseline aneurysm diameter and was only associated with the presence of peripheral artery disease (*Table 1*).

**Table 1:** Demographic details for the overall cohort and by classification categories for the MA^3^RS and SoFIA^3^ study cohorts

	Overall cohort (*n* = 338)	USPIO negative (*n* = 193)	USPIO positive (*n* = 145)	*P*	SoFIA^3^ cohort (*n* = 72)	Tertile 1 (*n* = 24)	Tertile 2 (*n* = 24)	Tertile 3 (*n* = 24)	*P*
Age (years), mean(s.d.)	73.5(7.2)	73.8(7.5)	73.2(6.8)	0.493	72.5(6.9)	73.3(7.2)	72.8(7.5)	71.4(6.1)	0.640
Sex—male	288 (85.2)	168 (87.0)	120 (82.8)	0.272	61 (85)	20 (83)	21 (88)	20 (83)	0.898
**Smoking**				<0.001*					0.365
Current	94 (27.8)	40 (20.7)	54 (37.2)		19 (26)	6 (25)	9 (38)	4 (17)	
Ex	195 (57.7)	122 (63.2)	73 (50.3)		47 (65)	17 (71)	12 (50)	18 (75)	
Never	46 (13.6)	31 (16)	15 (10.3)		6 (8)	1 (4)	3 (13)	2 (8)	
**Hypertension**	243 (71.9)	144 (74.6)	99 (68.3)	0.200	47 (65)	14 (58)	16 (67)	17 (71)	0.651
Systolic blood pressure (mmHg), mean(s.d.)	139.0(21.3)	140.1(21.3)	138.3(21.4)	0.425	136.7(18.3)	142.1(18.3)	132.5(16.2)	135.6(19.9)	0.178
Diabetes	47 (13.9)	31 (16.1)	16 (11.0)	0.186	10 (14)	3 (13)	4 (17)	3 (13)	0.890
Hypercholesterolaemia	256 (75.7)	147 (76.2)	109 (75.2)	0.833	59 (82)	21 (88)	21 (88)	17 (71)	0.223
Ischaemic heart disease	124 (36.7)	70 (36.3)	54 (37.2)	0.854	22 (31)	7 (29)	7 (29)	8 (33)	0.937
Peripheral artery disease	65 (19.2)	34 (17.6)	31 (21.4)	0.385	11 (15)	2 (8)	8 (33)	1 (4)	0.010*
Cerebrovascular disease	46 (13.6)	22 (11.4)	24 (16.6)	0.172	10 (14)	1 (4)	4 (17)	5 (21)	0.221
Family history of AAAs	62 (18.3)	34 (17.6)	28 (19.3)	0.690	9 (13)	2 (8)	4 (17)	3 (13)	0.683
Antiplatelet	222 (65.7)	127 (65.8)	95 (65.5)	0.956	51 (71)	19 (79)	18 (75)	14 (58)	0.244
Anticoagulant	24 (7.1)	15 (7.8)	9 (6.2)	0.579	2 (3)	1 (4)	0 (0)	1 (4)	0.598
Statin	265 (78.4)	152 (78.8)	113 (77.9)	0.855	58 (81)	21 (88)	21 (88)	16 (67)	0.109
Beta-blocker	75 (22.2)	44 (22.8)	31 (21.4)	0.756	19 (26)	8 (33)	5 (21)	6 (25)	0.606
ACE inhibitor	123 (36.4)	75 (38.9)	48 (33.1)	0.276	25 (35)	8 (33)	8 (33)	9 (38)	0.941
Aneurysm diameter at baseline (mm), mean(s.d.)	49.5(7.3)	48.2(6.7)	51.3(8.4)	<0.001*	48.8	47.5(9.2)	48.7(7.8)	50.1(5.8)	0.510

Values are *n* (%) unless otherwise indicated. *Statistically significant. MA^3^RS study = MRI using Ultrasmall Superparamagnetic Particles of Iron Oxide to Predict Clinical Outcomes in Patients Under Surveillance for Abdominal Aortic Aneurysms; ISRCTN76413758. SoFIA^3^ study = Sodium Fluoride Imaging of Abdominal Aortic Aneurysms; NCT02229006. USPIO negative = no enhancement of USPIO on MRI. USPIO positive = USPIO enhancement on MRI. Tertile 1 = low [^18^F]NaF uptake on PET/CT. Tertile 2 = moderate [^18^F]NaF uptake on PET/CT. Tertile 3 = high [^18^F]NaF uptake on PET/CT. USPIO, ultrasmall superparamagnetic particles of iron oxide; AAAs, abdominal aortic aneurysms; ACE, angiotensin converting enzyme; [^18^F]NaF, fluorine-18 sodium fluoride.

### Composite of rupture or repair

During follow-up, 212 patients (62.7%) in the overall cohort met the primary composite endpoint of aneurysm rupture (42 (12.4%), of whom 5 underwent emergency repair) or repair (176 (52.1%)) (*Table 2*).

**Table 2:** Clinical outcomes of patients overall and by classification group

	Overall (*n* = 338)	USPIO negative (*n* = 193)	USPIO positive (*n* = 145)	*P*	SoFIA^3^ cohort (*n* = 72)	Tertile 3 (*n* = 24)	Tertiles 1 and 2 (*n* = 48)	*P*	USPIO positive + tertile 3 (*n* = 12)	USPIO negative + tertile 1 or 2 (*n* = 60)	*P*
Aneurysm expansion (mm/year), median (i.q.r.)	2.9 (1.6–4.1)	2.5 (1.4–3.6)	3.3 (2.0–4.6)	0.002*	2.6 (1.3–4.1)	4.0 (2.6–5.4)	1.9 (0.9–3.0)	<0.001*	5.1 (3.0–6.2)	2.2 (1.2–3.6)	0.002*
**Composite of rupture or repair**	212 (62.7)	119 (61.7)	93 (64.1)	0.641	36 (50)	19 (79)	17 (35)	<0.001*	10 (83)	26 (43)	0.013*
Aneurysm repair	176 (52.1)	100 (51.8)	76 (52.4)	0.900	28 (39)	15 (63)	13 (28)	0.004*	7 (58)	21 (35)	0.130
Aneurysm rupture	42 (12.4)	21 (10.9)	21 (14.5)	0.299	8 (11)	4 (17)	4 (9)	0.336	3 (25)	5 (8)	0.111
Overall survival (months), median (i.q.r.)	102.2 (57.9–119.1)	106.4 (63.9–120.5)	99.6 (50.2–117.5)	0.084	101.4 (62.7–107.5)	102.9 (76–106.2)	101.2 (101.2–107.5)	0.364	99.3 (56–106.2)	101.8 (66.7–107.5)	0.613

Values are *n* (%) unless otherwise indicated. *Statistically significant. SoFIA^3^ study = Sodium Fluoride Imaging of Abdominal Aortic Aneurysms; NCT02229006. USPIO negative = no enhancement of USPIO on MRI. USPIO positive = USPIO enhancement on MRI. Tertile 1 = low [^18^F]NaF uptake on PET/CT. Tertile 2 = moderate [^18^F] NaF uptake on PET/CT. Tertile 3 = high [^18^F] NaF uptake on PET/CT. USPIO, ultrasmall superparamagnetic particles of iron oxide; i.q.r., interquartile range; [^18^F]NaF, fluorine-18 sodium fluoride.

There was no demonstrable association between baseline USPIO enhancement and future rupture or repair (log rank *P* = 0.13; *Fig. 2a*). During follow-up of 72 patients in the SoFIA^3^ study who underwent both USPIO-enhanced MRI and [^18^F]NaF PET/CT, 36 patients (50%) met the primary endpoint of rupture (8 patients (11%)) or repair (28 patients (39%)). Patients in the highest tertile of [^18^F]NaF uptake were twice as likely to experience rupture or repair (72% *versus* 36%; *P* = 0.002; *Fig. 2b*) and the association remained after adjustment for age, sex, current smoking, systolic blood pressure, and baseline aneurysm diameter (*P* = 0.003).

**Figure 2:**
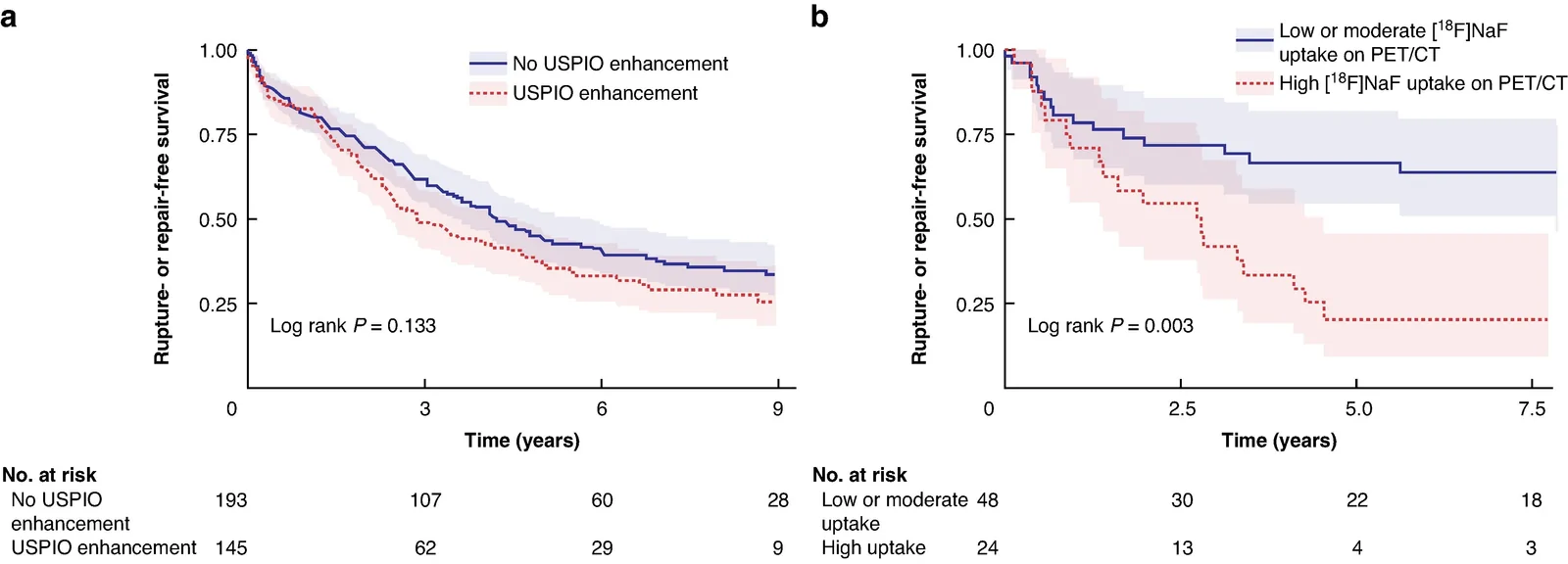
Survival analysis by groups **a** Repair- or rupture-free survival, according to aortic wall USPIO enhancement on MRI. **b** Repair- or rupture-free survival, according to aortic wall [^18^F]NaF uptake on PET/CT. USPIO, ultrasmall superparamagnetic particles of iron oxide; [^18^F]NaF, fluorine-18 sodium fluoride.

When evaluating the combination of USPIO MRI and [^18^F]NaF PET/CT in an exploratory analysis, those patients who had both USPIO enhancement and the highest tertile of [^18^F]NaF uptake were more likely to experience rupture or repair (10 of 12 patients (83%) *versus* 26 of 59 patients (44%); *P* = 0.013; *Table 2*) although this was of borderline statistical significance when adjusted for common risk factors for aneurysm rupture (*P* = 0.052).

### Aneurysm expansion

The median AAA expansion rate for the whole cohort was 2.9 (i.q.r. 1.6–4.1) mm/year, which was similar to that for the SoFIA^3^ substudy cohort (2.6 (i.q.r. 1.3–4.1) mm/year). In unadjusted analysis, USPIO enhancement was associated with future greater aneurysm expansion (3.3 (i.q.r. 2.0–4.6) mm/year, compared with 2.5 (i.q.r.1.4–3.6) mm/year without USPIO enhancement; *P* = 0.002). This association persisted after adjustment for current smoking, hypertension, and baseline aneurysm diameter (β coefficient = 0.72 (95% c.i. 0.04 to 1.40) mm/year; *P* = 0.038), although the model fit was weak (R^2^ = 0.051, adjusted R^2^ = 0.037; *Table 3*).

**Table 3:** Multivariable linear regression analysis of the association between USPIO enhancement on MRI and AAA expansion

Model	Coefficients					
	Unstandardized	s.e.	*t*	*P*	95% c.i.	
					Lower	Upper
M_1 (intercept)_	0.39	1.26	0.31	0.759	−2.01	2.87
Current smoker (yes)	0.33	0.37	0.90	0.367	−0.40	1.06
USPIO enhancement (yes)	0.72	0.35	2.08	0.038	0.04	1.40
Baseline AAA size (mm)	0.06	0.03	2.12	0.035	0.04	0.11
Hypertension (yes)	0.42	0.36	1.17	0.244	−0.29	1.14

Model summary (rate of AAA expansion (mm/year)): M_1_—R = 0.225, R^2^ = 0.051, adjusted R^2^ = 0.037. M_1_ includes USPIO enhancement, current smoker, hypertension, and baseline AAA size. AAA, abdominal aortic aneurysm; USPIO, ultrasmall superparamagnetic particles of iron oxide.

Patients in the highest tertile of [^18^F]NaF uptake had an aneurysm expansion rate more than twice as high as those in the lowest or intermediate tertile in unadjusted analysis (median of 4.0 (i.q.r. 2.6–5.4) *versus* 1.9 (i.q.r. 0.9–3.0) mm/year; *P* < 0.001). In a linear regression analysis, high uptake was independently associated with future expansion, even when adjusted for current smoking, hypertension, and baseline aneurysm diameter (β coefficient = 1.7 (95% c.i. 0.6 to 2.8) mm/year; *P* = 0.003). Baseline size and hypertension were also important determinants of expansion (*Table 4*).

**Table 4:** Multivariable analysis of the association between [^18^F]NaF uptake on PET/CT and AAA expansion

Model	Coefficients					
	Unstandardized	s.e.	*t*	*P*	95% c.i.	
					Lower	upper
M_1 (intercept)_	−4.48	2.2	−2.07	0.043	−8.8	−0.1
Current smoker (yes)	0.42	0.6	0.74	0.460	−0.7	1.5
[^18^F]NaF uptake (high)	1.68	0.5	3.11	0.003	0.6	2.8
Baseline AAA size (mm)	0.14	0.05	3.1	0.003	0.1	0.2
Hypertension (yes)	1.51	0.5	0.50	0.004	0.5	2.5

Model summary (rate of AAA expansion (mm/year)): M_1_—R = 0.621, R^2^ = 0.386, adjusted R^2^ = 0.341. M_1_ includes current smoker, high [^18^F]NaF uptake, baseline AAA size (mm), and hypertension status. AAA, abdominal aortic aneurysm; [^18^F]NaF, fluorine-18 sodium fluoride.

In an exploratory analysis, the combination of USPIO enhancement and the highest tertile of [^18^F]NaF uptake was associated with the greatest aneurysm expansion rate (median of 5.1 (i.q.r. 3.0–6.2) mm/year) and was higher than that for the highest tertile of [^18^F]NaF uptake alone (median of 4.0 (i.q.r. 2.6–5.4) mm/year) and more than twice as high as that for no USPIO enhancement and low or moderate [^18^F]NaF uptake (median of 2.6 (i.q.r. 1.2–3.6) mm/year; *P* = 0.002; *Fig. 3*), even when adjusted for hypertension, current smoking, and baseline aneurysm diameter (β coefficient = 1.53 (95% c.i. 0.06 to 3.01) mm/year; *P* = 0.041).

**Figure 3:**
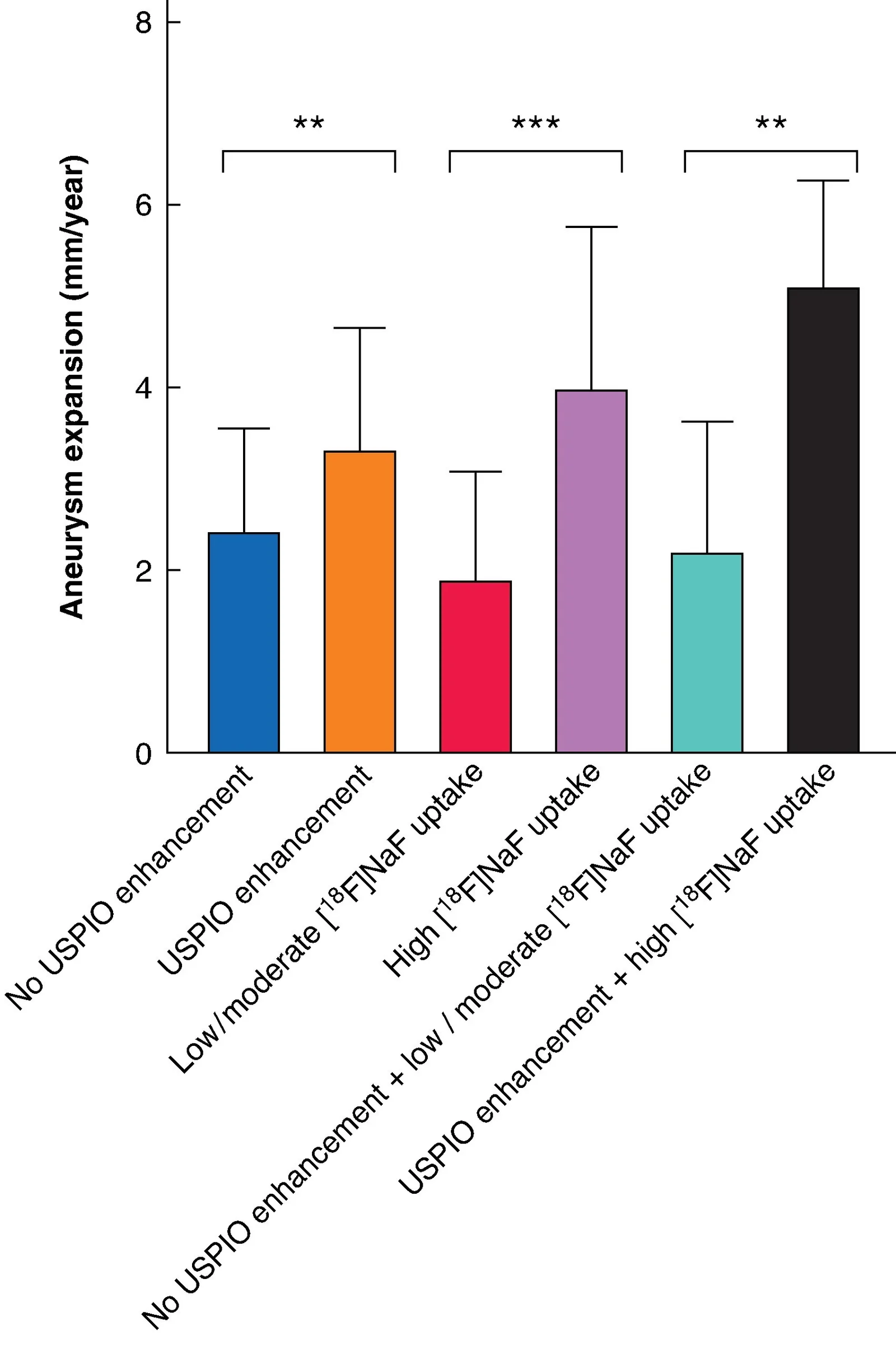
Aneurysm expansion rate according to imaging group Median (i.q.r.) aneurysm expansion (mm/year) in groups stratified by aortic wall biological activity, as characterized using USPIO MRI and [^18^F]NaF PET/CT. i.q.r., interquartile range; USPIO, ultrasmall superparamagnetic particles of iron oxide; [^18^F]NaF, fluorine-18 sodium fluoride. **P=0.002; ***P < 0.001

### Survival

Overall, 193 patients (57.1%) died, of whom 45 (23.3%) had aneurysm-related deaths and 43 (22.3%) had other cardiovascular deaths (*Table 5*). Of those patients with USPIO enhancement on MRI (145 of 338 patients (42.9%)), 91 (62.8%) died during follow-up, which appeared to be higher than for those without USPIO enhancement (102 of 193 patients (52.8%)), but this did not reach statistical significance (*P* = 0.068). Median survival appeared to be longer in patients with no USPIO enhancement (3398 *versus* 2989 days; log rank *P* = 0.085), but again was not statistically significant. USPIO enhancement was not associated with aneurysm-related (log rank *P* = 0.098) or cardiovascular (*P* = 0.422) death.

**Table 5:** Causes of death, as determined through review of medical records and death certificates; *n* = 193

Category (*n*)	Subcategory (*n*)	Specific cause of death
Cardiovascular death—non-AAA-related (43)	Pulmonary oedema (7)	N/A
	Stroke (10)	N/A
	Myocardial infarction/ischaemic heart disease (19)	N/A
	Other cardiovascular (7)	Dementia (2) Mesenteric ischaemia (1) Chronic limb threatening ischaemia (2) Other (2)
Cardiovascular death—AAA-related (45)	Ruptured AAA (42)	N/A
	AAA repair complications (3)	Aorto-duodenal fistula (1) Mesenteric ischaemia (1) Aortic dissection (1)
Non-cardiovascular death (75)	Malignancy (28)	N/A
	Lower respiratory tract infection (24)	N/A
	Chronic obstructive pulmonary disease (2)	N/A
	COVID (3)	N/A
	Other (18)	Acute kidney injury (1) Frailty (2) Bowel perforation (1) Interstitial lung disease (2) Pancreatitis (2) Diverticulitis (1) Sepsis (2) Subdural haematoma (1) Gastrointestinal haemorrhage (2) Not specified (4)
Unknown (30)	N/A	N/A

AAA, abdominal aortic aneurysm.

Neither [^18^F]NaF uptake nor the combination of USPIO enhancement and high [^18^F]NaF uptake (*P* > 0.05 for all) was associated with overall survival, aneurysm-related death, or cardiovascular death.

## Discussion

During long-term follow-up of patients with asymptomatic AAAs enrolled in two prospective molecular and cellular imaging studies^7,15^, aortic wall macrophage-mediated inflammation was evaluated (using USPIO-enhanced MRI) and microcalcification activity was evaluated (using [^18^F]NaF PET/CT). The most biologically active aneurysms were more vulnerable to AAA expansion, independent of clinical risk factors. In an exploratory analysis, the combination of aortic wall USPIO enhancement on MRI and high [^18^F]NaF uptake on PET/CT was associated with increased aneurysm expansion. Aneurysms with high [^18^F]NaF uptake alone were more than twice as likely to rupture or need repair, independent of clinical risk factors. This suggests that both USPIO enhancement and [^18^F]NaF uptake are important imaging biomarkers, identifying distinct, but additive, processes related to AAA disease progression that together may identify the highest-risk aneurysms.

The value of imaging biomarkers lies in their ability to enable understanding of the biology of the aortic wall on a molecular and cellular level without an invasive biopsy, meaning that disease activity can be evaluated at an early disease stage in advance of major clinical events. Whilst there are multiple pathobiological processes implicated in aneurysm formation and progression, this study investigated microcalcification and macrophage activity using two molecular and cellular imaging probes that are known to be safe with good reproducibility and robust pilot data^6,12^. The synergistic effect of these processes is incompletely understood, but they ultimately coalesce to degrade the extracellular matrix, leading to aortic wall degeneration and dilatation. Inflammation is a key factor, especially in the early stages of AAA initiation; macrophage infiltration into the adventitia mediates an inflammatory tissue response, producing cytokines and chemokines leading to loss of structural wall integrity^19^. This may explain why USPIO enhancement was associated with baseline aneurysm diameter, perhaps representing cumulative structural changes as a result of tissue-resident macrophage activity. Cells within the aortic wall also undergo maladaptive transformation and, in particular, VSMC death. Cellular phenotype switching leads to loss of adaptive aortic wall function, further inflammation, and the laying down of hydroxyapatite to form foci of microcalcification activity^20^. In this cohort, microcalcification activity was not correlated with baseline AAA size, suggesting that this pathway may be more reflective of dynamic and active destabilization and remodelling following aneurysm initiation. These two target-specific imaging probes can quantitatively evaluate the disease processes underpinning this complex inflammatory disease process and the data suggest that the evaluation of multiple processes simultaneously may provide the best predictive value.

This imaging approach is highly relevant in AAA disease management, as it is widely recognized that aneurysm expansion and rupture risk cannot be accurately predicted on an independent patient level. Whilst baseline aneurysm diameter is the most common clinical risk factor associated with future disease progression, it is an imperfect parameter on which to base management of the disease. The present study findings, and those of others^21^, suggest that biological activity in the wall of AAAs can provide important prognostic data in addition to clinical features. The most commonly evaluated imaging biomarkers in aortic disease to date have targeted molecular elements of the inflammatory process. [^18^F]FDG identifies focal areas of increased glucose metabolism and has had some promising^22,23^, but conflicting^8–10^, results in patients with AAAs. [^18^F]NaF identifies the earliest part of the vascular calcification process and has also been used to evaluate other aortic pathologies with success^24–26^. Both radiotracers, and others^27–29^, have been used in atherosclerotic disease elsewhere^18,30,31^, but none has been adopted into routine clinical practice.

USPIO MRI has been evaluated in the aorta^7,23,32^ and carotid arteries^4,5,33^ and, whilst the image analysis is complex and time-consuming, it has also shown promise as a surrogate endpoint in drug trials^34^. Other biomarkers, such as those found in serum or plasma, have been investigated in AAA disease with some success^35–37^, but circulating blood biomarkers enable systemic evaluation, whereas imaging techniques can visualize, localize, and quantify aortic tissue-specific biological activity.

The authors acknowledge some key limitations. The USPIO agent used in the MA^3^RS study (ferumoxytol) remains off label for clinical imaging purposes and the formulation used in the study (Rienso, Takeda) is no longer available in Europe. Whilst there have been safety concerns about rare, but serious, acute hypersensitivity reactions caused by ferumoxytol, potentially limiting its use as an imaging contrast agent, there were no serious adverse effects noted during the trial. In addition, international multicentre data suggest that it is also well tolerated, with a positive safety profile when used off label for diagnostic use^38^. Generic USPIO agents remain available and approved for use in iron-deficiency anaemia, but are unlikely to be made widely available to patients with AAAs. The use of PET/CT is expensive, involves ionizing radiation, and is not readily available in all sites. However, many patients with cancer undergo PET/CT as part of routine assessment and it is suggested that the cost of unnecessary aneurysm repair, surveillance, or life years lost could be weighed up against the cost of additional imaging in a selected group of patients.

This study evaluated the combination of USPIO MRI and [^18^F]NaF PET/CT as an exploratory analysis, recognizing that the median time between scans was 14 months and that the numbers in each group were small, with the analysis therefore vulnerable to type II error. This may limit interpretation when combining these two imaging probes, but remains an interesting first step in multiprobe disease evaluation. Reassuringly, the long-term results presented in this study are consistent with previous findings that both imaging probes can predict aneurysm disease activity in the medium-term, suggesting that it may be possible to detect a distinct individual biological signature that persists over time. Future studies evaluating the combination of molecular and cellular imaging targets using different imaging probes should aim to undertake these scans contemporaneously and to account for the difference in spatial resolution between MRI and PET/CT. In the present study, it was not possible to co-register the MRI and PET/CT data in order to determine the anatomic co-location of inflammation and microcalcification, but this should be considered for future studies.

Some death certificates were found to be unavailable due to delays in updating the public record system and the very low rate of post-mortem examination in the UK is also recognized, which means that the interpretation of death certificate data must be taken with caution, but is a universal problem in such studies. The use of a composite endpoint has limitations, but is common in clinical trials where the event rate (such as aneurysm rupture) is low and would require a much larger population to achieve adequate power. The decision to undertake aneurysm repair is based on more than just size, and this must be taken into account when considering the pragmatic composite clinical endpoint.

This study remains the largest prospective study of molecular or cellular imaging of AAAs to date, with long-term progression data. In addition, it is the first to explore the combination of aortic wall USPIO enhancement and [^18^F]NaF uptake in patients with AAAs. High aortic wall [^18^F]NaF uptake emerges as the most promising imaging biomarker of future aneurysm events to date. This is an exciting development in the field of cardiovascular imaging and may offer new avenues for personalized medicine in vascular disease by tailoring management and surveillance intervals to aneurysm-specific biology. Specifically, this approach has the potential to be used for patients in whom the treatment decision (repair *versus* ongoing surveillance) is not straightforward. This includes patients who have large aneurysms and co-morbidities where the benefit of intervention is uncertain, and it may have a role in refining and personalizing surveillance programmes to patients according to their biological risk in order to target intervention in a timely manner.

There is also great potential to use imaging biomarkers in therapeutic trials, allowing mechanistic evaluation of drug therapies or using imaging endpoints as surrogates for clinical outcomes that would otherwise take many years to become evident. The identification of a unique biological signature using these non-invasive molecular imaging techniques may help guide personalized management of patients with AAAs.

## Data Availability

Data are available from the corresponding author upon request.
